# Efficacy of providing energy expenditure information to guide weight loss interventions in people with obesity: A randomized controlled trial

**DOI:** 10.1111/cob.12703

**Published:** 2024-09-17

**Authors:** Jonathan Z. M. Lim, Andrew Williams, Jamie Burgess, James O'Connell, Michaela James, Andy Cross, David Hughes, Daniel J. Cuthbertson, Uazman Alam, John P. H. Wilding

**Affiliations:** ^1^ Department of Cardiovascular & Metabolic Medicine Institute of Life Course and Medical Sciences, Clinical Sciences Centre, Liverpool Centre for Cardiovascular Science, University of Liverpool Liverpool UK; ^2^ Diabetes, Endocrinology, and Metabolism Centre, Manchester Royal Infirmary, Manchester Academic Health Science Centre Manchester University NHS Foundation Trust Manchester UK; ^3^ Institute of Cardiovascular Sciences, Cardiac Centre, Faculty of Medical and Human Sciences University of Manchester and NIHR/Wellcome Trust Clinical Research Facility Manchester UK; ^4^ Aintree Weight Management Services Nutrition and Dietetics Therapies, Aintree University Hospital, Liverpool University Hospitals NHS Foundation Trust Liverpool UK; ^5^ Department of Health Data Science Institute of Population Health, Faculty of Health & Life Sciences, University of Liverpool Liverpool UK; ^6^ Centre for Biomechanics and Rehabilitation Technologies Staffordshire University Stoke‐on‐Trent UK

**Keywords:** diet, energy expenditure, exercise, fat oxidation, indirect calorimetry, obesity

## Abstract

Resting energy expenditure (REE) and metabolic fuel utilization (carbohydrate or fat) proxied by respiratory quotient (RQ) from indirect calorimetry enables more precise measurement of energy needs and fat oxidation capacity. The study compared the effectiveness of providing energy expenditure information during diet and exercise weight intervention versus standard of care (SOC) on weight loss outcomes. Fifty‐two participants with obesity were recruited from a specialist weight loss service, randomized 1:1 to intervention (INT) or SOC only. Participants in INT received four‐weekly dietetic counselling, using biofeedback from energy expenditure data to recommend caloric restriction and physical activity goals, in addition to SOC. The primary outcome was the mean difference in weight loss between both groups after 24 weeks. Secondary outcomes include participant acceptability and tolerability using indirect calorimetry. Participants in the INT group demonstrated additional weight loss (−2.3 kg [95% CI: −3.1, −1.5]; *p* <.001), reduced waist circumference (−3.9 cm [95% CI: −5.48, −2.26]; *p* <.001), and decreased body fat percentage (−1.5% [95% CI:−2.31, −0.72], *p* <.001), compared to SOC, after adjusting for baseline body mass index, age, and sex. Forty‐two percent (10/24) of participants in INT group achieved the minimum clinically significant threshold of 5% weight loss from baseline, compared to 8% (2/26) in the SOC group (*p* = .007). Participant acceptability and tolerability of indirect calorimetry were high, with mean scores of 4.5 ± 0.6 and 4.2 ± 0.7 (5‐point Likert scale). The study establishes the safety and practical integration of biofeedback using indirect calorimetry promoting improved self‐regulation and enhancing weight loss.

## Graphical abstract

Providing Energy Expenditure Biofeedback to Guide Weight Loss Intervention in Individuals with Obesity resulted in additional weight loss compared to standard of care.

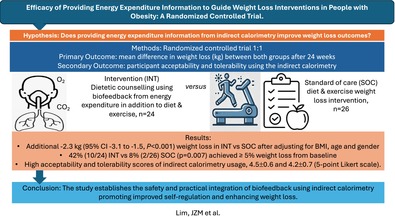

## 1 INTRODUCTION

Obesity is a chronic disease strongly associated with multiple cardiovascular and metabolic complications.^1^, ^2^ Globally the rise in obesity has occurred very rapidly with an increase of 30–50% per decade in high‐income countries.^3^ Coping with the recommended lifestyle change has proven to be remarkably challenging and most lifestyle interventions have been shown to induce only modest, and often non‐sustained, changes in target behaviours. Combination of diet, physical activity, and behavioural interventions on average achieves 2%–5% weight loss with the additional cardiovascular benefits of prevention of obesity‐related comorbidities.^4^ However, many have trouble maintaining and adhering to lifestyle interventions leading to limited weight loss.

During periods of energy restriction, compensatory metabolic and behavioural responses often occur, which may attenuate the effects of the prescribed energy deficit. These responses include reduced resting energy expenditure (REE), increased hunger, and alterations in circulating levels of hormones involved in appetite regulation that promote weight regain.^5^ The respiratory quotient (RQ) is a valuable metric, representing the ratio of carbon dioxide produced to oxygen consumed (VCO_2_/VO_2_) during substrate metabolism. An RQ value provides insights into which macronutrients (carbohydrates, fats, or proteins) are being oxidized to meet energy demands. An RQ of 1.0, 0.8, and 0.7 is consistent with carbohydrate, protein, and fat oxidation respectively.^6^ Thus, a lower RQ implies a metabolic shift towards increased fat oxidation.^7^

The rationale for this study was to test whether providing people with obesity with personalized energy expenditure data using a portable indirect calorimetry (IC), could improve weight loss outcomes. Utilizing biofeedback from energy expenditure data (REE and RQ) helps inform individuals about their energy needs and capacity for fat oxidation.^8^

## 2 OBJECTIVES

### 2.1 Primary objective

The primary aim was to compare the effectiveness of providing energy expenditure information to both clinicians and participants in addition to standard of care (SOC) versus SOC only on weight loss in a secondary care‐based specialist weight management service (SWMS).

## 3 METHODOLOGY

### 3.1 Study design and participants

**FIGURE 1:**
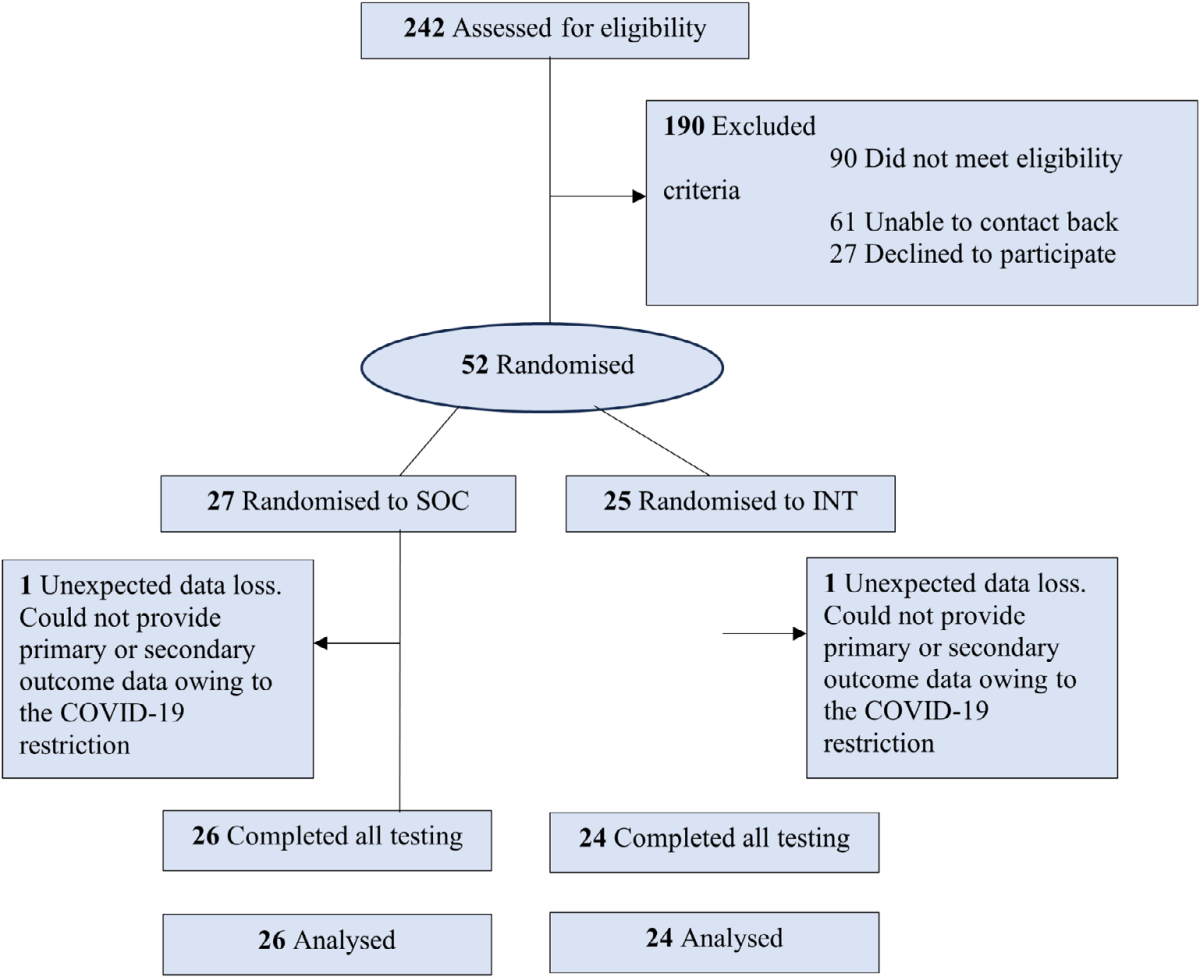
Participant recruitment flow diagram. INT, intervention group; SOC, standard care group.

In the United Kingdom, the ‘tier 3’ SWMS are multidisciplinary programs designed for individuals with obesity and related health issues, offering personalized care including medical, nutritional, and psychological support.^9^ Participants with obesity attending the specialist weight management clinic in the Liverpool University Hospitals NHS Foundation Trust were recruited between December 2018 and February 2022 by direct email, clinic newsletter, and physician referral. Participants were eligible if they had a body mass index (BMI) ≥30 kg/m,^2^ attending the SWMS,^4^ and did not have diabetes or a severe or unstable chronic medical condition.^10^ We screened 242 people and enrolled 52 participants (Figure 1). All participants provided written informed consent and the study was approved by the Health Research Authority, North‐West Research Ethics Committee (REC 18/NE/0645). The design, conduct, and reporting of this study were approved and sponsored by the University of Liverpool/ Liverpool Joint Research Office. The study protocol and planned statistical analysis have previously been reported.

### 3.2 Intervention group and randomization

The ECAL study (NCT03638895) was a 24‐week assessor‐blinded, parallel‐arm, randomized controlled weight‐loss trial. Eligible participants were randomized to receive EE data from the indirect calorimetry‐guided intervention (INT) or the SOC group. The study was assessor‐blinded, and participants were informed of their group assignment at baseline. Randomization was performed by the statistician in a 1:1 allocation ratio, with stratification by sex and BMI, using the RedCap randomization module.

### 3.3 REE measurement

To measure REE using the portable ECAL IC (Metabolic Health Solutions Pty Ltd, Australia), participants were required to fast overnight, abstain from caffeine and alcohol for at least 12 hours, and avoid strenuous exercise for 24 h prior to undergoing IC measurements. REE was measured in a thermoneutral environment while donning a nose clip and breathing through a single‐use mouthpiece, obtained whilst resting in a supine position. To ensure a steady state, the first 10 min of oxygen consumption (VO_2_) and carbon dioxide production (VCO_2_) were discarded, and the remaining 20 min were used to confirm stable oxygen and carbon dioxide measurements. Upon completion of IC measurement, the ECAL IC in‐built software generates a layperson summary with REE and RQ data. The layperson summary or report was provided to both participants and registered dietitians providing 4‐weekly dietetic counselling to participants in the INT group. An example of this layperson summary can be visualized in Supplementary Material. Although participants in the SOC group underwent the ECAL IC measurements, neither they nor the treating clinicians received any information about their measured energy expenditure data.

### 3.4 Dietetic counselling using energy expenditure information

All participants received one‐to‐one dietetic counselling from a registered dietitian at baseline (60‐min session), followed by 4‐weekly 30‐min dietetic counselling sessions over the 24‐week intervention. All participants were encouraged to maintain the recommended caloric restriction (minimum of 30% energy restriction or 500 kcal/day below their total daily energy needs, whichever was the greater of the two calculations) and increase their physical activity (aiming for a minimum of 150 min of physical activity per week) throughout the 24‐week duration.

Dietitians utilized information about energy requirements, and carbohydrate or fat oxidation capacity which was communicated in the form of a layperson summary (Supplementary Material). The biofeedback from energy expenditure data was used to recommend personalized calorie restrictions and review the participants' self‐reported food diaries, offering suggestions or recommending changes to their weekly meal plans. Participants in the INT group received ongoing feedback based on their progress, which was monitored through regular four‐weekly REE measurements to ensure sustained energy restriction. Counsellors used this information to make real‐time adjustments to dietary and physical activity recommendations to align with the participants' energy expenditure and weight loss goals.

In comparison, participants in the SOC group received standard dietary counselling without the integration of biofeedback from indirect calorimetry. SOC group had similar caloric goals based on their calculated total daily energy needs using the Harris–Benedict formula^11^ and similar recommendations for physical activity goals. They attended the same number of dietetic counselling sessions but without personalized adjustments based on energy expenditure data.

### 3.5 Outcome measures

#### 3.5.1 Primary outcome measure

The primary outcome was the mean difference in body weight (kg) between INT and SOC groups after 24 weeks from baseline.

#### 3.5.2 Secondary outcome measures

The secondary outcome was the participant's acceptability and tolerability of utilizing the portable ECAL IC. Other secondary outcomes included the change in REE and RQ during the dynamic phase of weight loss (weeks 4 and 12) and at end of study (week 24).

### 3.6 Body composition

Body composition was measured using the TANITA (TBF‐300MA, Tanita, Tokyo, Japan) two‐electrode leg‐to‐leg bioimpedance analyser. Weight and height were obtained on a calibrated digital scale and wall‐mounted stadiometer. BMI was calculated as weight (kg)/height^2^(m).

### 3.7 Dietary analysis

Participants reported what they have eaten, and self‐reported the amount, volumes, and portion sizes for each meal through paper or electronic food diaries (at least 2 weekdays and 1 weekend day). Self‐reported energy intake (kcal/day) was calculated and compared between baseline, intervals (weeks 4, 8, and 12), and final visit. Participants were classified as adherent if they followed the recommended energy restriction for at least 70% of the time. Days with missing surveys were considered nonadherent.

### 3.8 Physical activity analysis

Activity‐related energy expenditure (AEE) and physical activity duration were measured using the International Physical Activity Questionnaire (IPAQ). Data from the IPAQ was utilized to calculate the metabolic equivalents (METS) expressed in MET‐min/week.

### 3.9 Patient acceptability and tolerability questionnaires

Patients were asked to complete a few 5‐point Likert scale questionnaires to measure acceptability and tolerability of using the portable ECAL IC, with responses ranging from ‘strongly disagree’ (1) to ‘strongly agree’ (5) at the beginning and end of the study. These questions include ease of use, comfort, effectiveness, tolerability, overall satisfaction, and whether they would recommend the use of the device to others. Mean scores were averaged at the end of the 24‐week study.

### 3.10 Power calculation

The trial was statistically powered to detect a between‐group difference of 3 kg. Based on a retrospective dataset with a standard deviation of 4 kg, 42 participants per group were needed to achieve 80% power to detect a 3 kg between‐group difference at a 5% significance level using a two‐sided test. Accounting for a 20% attrition rate, the target recruitment was 102 participants. The study achieved 62% (52/84) of required sample size. Due to the COVID‐19 pandemic, further recruitment of participants was hindered. Fifty participants completed all aspects of the study.

## 4 STATISTICAL ANALYSIS

Continuous variables were evaluated for normality of distribution using visual inspection of histogram and the Shapiro Wilks test. All variables are presented as mean ± SD for normally distributed variables. Data from non‐normally distributed variables are presented as median and inter‐quartile range (IQR). The one‐way repeated measures ANCOVA was performed to assess the impact of weight change (primary outcome) while controlling for baseline BMI, age, and gender. Fisher's exact test was used to compare the proportion of participants who achieved the 5% weight loss threshold between both groups. For secondary analysis, ANCOVA was performed to investigate the change in RQ, REE, and body fat percentage. If the overall ANCOVA model was significant, post‐hoc tests (pairwise comparisons with Bonferroni correction) were conducted to identify specific differences between groups at each time point (4, 12, and 24 weeks). We used paired sample *t*‐tests to analyse post‐hoc differences at specific time points (4, 12, and 24 weeks), comparing participants' measurements before and after intervention at each time point to assess changes within the same group over time. For evaluating general trends, we conducted repeated measures analyses, with Greenhouse–Geisser correction. Analyses were performed in SPSS, version 27 (SPSS Inc., Chicago, Iln, USA) using two‐sided tests with α = .05. All analyses were intention‐to‐treat, except that the food adherence surveys and questionnaire data were analysed in completers only.

## 5 RESULTS

### 5.1 Participant characteristics

Fifty‐two participants were randomized, but only 50 people completed all aspects of the study. Two participants withdrew because they were unable to provide primary or secondary outcome data owing to the COVID‐19 restrictions. Participants had a mean (SD) BMI of 41.6 (2.6) kg/m^2^ and a mean age of 46.7 (10.4) years. Forty‐one participants (82%) were female; 8 (16%) were Asian, 12 (24%) were Black, and 30 (60%) were White. No adverse events were reported. Baseline demographics and characteristics, body composition, biochemistry tests, and energy expenditure data are summarized in Table 1. The cohort of patients had multiple comorbidities including hypertension (65%), sleep apnoea (50%), non‐alcoholic fatty liver disease (48%), and hyperlipidaemia (67%). The study excluded participants with type 2 diabetes. Across a total of seven scheduled study visits, participants attended an average of 5.7 (±0.5) and 6.0 (±0.7) study visits in the SOC and INT groups respectively. Attendance rates did not differ between groups (*p* = .79).

**TABLE 1:** Baseline characteristics of the intention‐to‐treat population.

	SOC (*n* = 27)	INT (*n* = 25)	All participants (*n* = 52)
Age, years	48.0 (10.6)	45.3 (10.1)	46.7 (10.4)
Gender			
Female, no. (%)	22 (81)	19 (76)	41 (79)
Male, no. (%)	5 (19)	6 (24)	11 (21)
Anthropometric data			
Body weight, kg	112.1 (8.1)	113.5 (7.5)	112.8 (7.8)
BMI, kg/m^2^	41.95 (2.21)	41.40 (2.70)	41.68 (2.45)
Waist circ., cm	118.4 (6.3)	116.3 (7.3)	117.4 (6.8)
Waist‐to‐hip ratio	0.95 (0.03)	0.96 (0.04)	0.96 (0.04)
Body fat, %	48.0 (2.0)	47.4 (2.1)	47.7 (2.0)
Fat mass, kg	54.9 (7.3)	54.3 (7.0)	54.6 (7.1)
Fat‐free mass, kg	59.3 (5.7)	60.1 (6.5)	59.7 (6.1)
Systolic BP, mmHg	136 (17)	133 (16)	134 (16)
Diastolic BP, mmHg	76 (12)	80 (10)	78 (11)
Comorbidities			
Hypertension, no. (%)	18 (66)	16 (64)	34 (65)
Sleep Apnoea, no. (%)	14 (52)	12 (48)	26 (50)
NAFLD, no. (%)	12 (46)	13 (52)	25 (48)
Hyperlipidaemia, no. (%)	18 (67)	17 (68)	35 (67)
Indirect calorimetry data			
Measured REE, kcal/day	1779 (131)	1749 (132)	1765 (131)
Predicted REE^a^, kcal/day	1756 (164)	1737 (101)	1747 (137)
Respiratory quotient (RQ)	0.972 (0.027)	0.983 (0.026)	0.977 (0.027)
Fat burn, %	3.3 (5.6)	1.6 (3.4)	2.5 (4.7)
Glucose availability, %	96.7 (5.6)	98.4 (3.4)	97.5 (4.7)
Diet and physical activity			
Total daily energy intake, kcal/day	1817 (190)	1845 (186)	1830 (187)
IPAQ‐S walking, min/week	245 (44)	224 (62)	235 (54)
IPAQ‐S moderate‐Int., min/week	114 (29)	111 (26)	112 (28)
IPAQ‐S vigorous‐Int., min/week	14 (11)	17 (11)	16 (11)
Total MET‐minutes/week	2125 (376)	2016 (392)	2072 (384)
Biochemistry			
HbA1c, mmol/mol	37.4 (2.8)	38.6 (2.5)	37.8 (2.8)
Fasting Glucose, mmol/L	5.0 (0.5)	5.1 (0.5)	5.1 (0.5)
Total cholesterol, mmol/L	5.20 (1.03)	5.45 (0.90)	5.32 (0.97)
HDL‐c, mmol/L	1.20 (0.26)	1.28 (0.32)	1.24 (0.29)
LDL‐c, mmol/L	3.05 (0.93)	3.41 (0.75)	3.21 (0.87)
Triglycerides, mmol/L	2.08 (1.26)	2.07 (0.95)	2.07 (1.12)
Creatinine mmol/L	68 (11)	71 (14)	69 (13)
eGFR ml/min/1.73 m^2^	85 (7)	79 (13)	83 (11)
Bilirubin, μmol/L	7 (3)	9 (4)	8 (4)
Albumin, g/L	44 (3)	44 (2)	44 (2)
ALT, IU/L	27 (18)	30 (18)	28 (18)
ALP, IU/L	89 (20)	85 (28)	87 (24)
AST, IU/L	22 (13)	22 (9)	22 (11)

*Note*: Values are expressed as mean (standard deviation) or number of participants (%).

Abbreviations: ALT, alanine aminotransferase liver enzyme; ALP, alkaline phosphatase liver enzyme; AST, aspartate aminotransferase liver enzyme; BMI, body mass index; eGFR, estimated glomerular filtration rate; FM, fat mass; FFM, fat‐free mass; HbA1c, glycated haemoglobin; HDL‐C, HDL‐cholesterol; INT, intervention group; IPAQ‐S, international Physical Activity Questionnaire‐Short Form; LDL‐C, LDL‐cholesterol; REE, resting energy expenditure; RQ, respiratory quotient; SOC, standard care group; TC, total cholesterol; WC, waist circumference.

^a^ Predicted Resting Energy Expenditure calculated based upon the Harris–Benedict equation.

**FIGURE 2:**
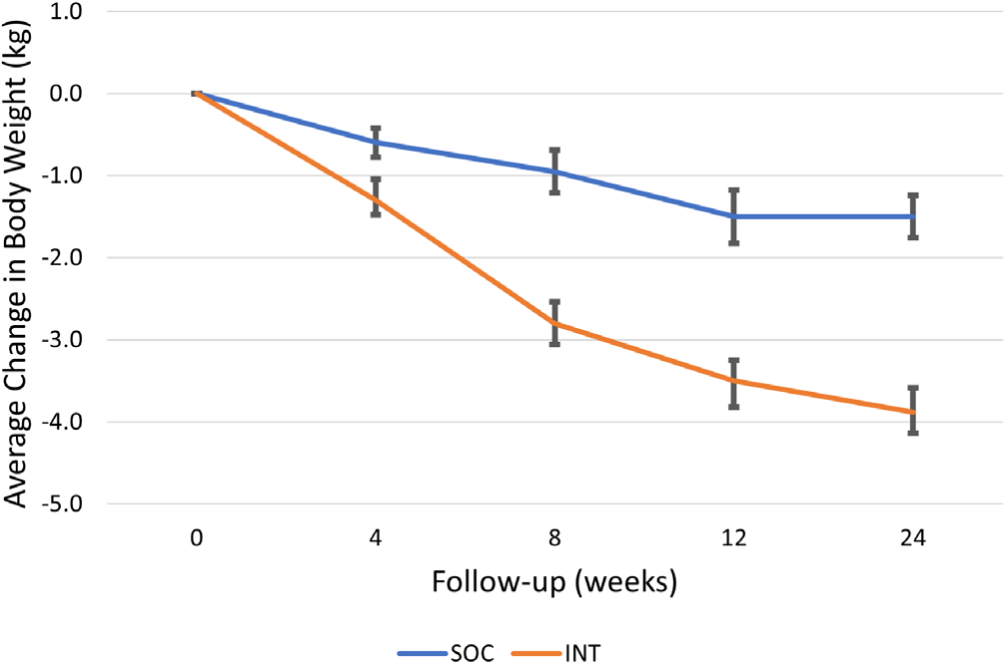
Mean change in body weight between INT and SOC. Error bars represent standard error.

After the 24‐week intervention, the INT group lost an additional −2.3 kg (95% CI: −3.1 to −1.5, *p* <.001) body weight as compared to SOC, after adjusting for baseline BMI, age, and gender (Table 2 and Figure 2). There were no differences in the changes in fat mass (*p* = .136), fat‐free mass (FFM; *p* = .478), and waist circumference between groups. For both within‐group comparisons, there was a decrease in FFM in both SOC (−1.3 kg; 95% CI: −2.3 to −0.4 kg, *p* = .007) and INT (−2.0 kg; 95% CI: −3.2 to −0.8 kg, *p* = .002) groups after 24‐weeks follow‐up (Table 3). Our results show that a greater proportion of the INT group (10/24 participants (42%)) achieved the minimum clinically significant threshold of 5% weight loss from baseline body weight, as compared to the SOC group (2/26 participants (8%); *p* = .007).

**TABLE 2:** Analysis of covariance (ANCOVA) of the mean difference in outcome measures between INT versus SOC.

	Mean difference	95% CI	*p‐*value
Weight loss (kg)^a^	−2.29	−3.07; −1.51	<.001
Percent weight loss (%)^a^	−2.01	−2.70; −1.32	<.001
Waist circumference (cm)^b^	−3.87	−5.48; −2.26	<.001
Body fat (%)^c^	−1.52	−2.31; −0.72	<.001
Resting energy expenditure (kcal/day)^d^	−36.6	−69.5; −4.3	.028
Respiratory quotient^e^	−0.04	−0.09; 0.01	.152
Self‐reported energy intake (kcal/day)^f^	−168	−263; −73	<.001

^a^ Adjusted for group, baseline BMI, age, and gender.

^b^ Adjusted for group, baseline BMI, baseline waist circumference, age, and gender.

^c^ Adjusted for group, baseline BMI, baseline body fat percentage, age, and gender.

^d^ Adjusted for group, baseline BMI, baseline REE, age and gender.

^e^ Adjusted for group, baseline BMI, baseline RQ, age and gender.

^f^ Adjusted for group, baseline BMI, baseline energy intake, age and gender.

**TABLE 3:** Mean differences in anthropometric, metabolic, and energy expenditure variables between baseline and week 24.

	SOC, *n* = 26			INT, *n* = 24			
	MD	95% CI	*p*‐value (W0 vs. W24)	MD	95% CI	*p*‐value (W0 vs. W24)	*p*‐value (SOC vs. INT)
Weight loss (kg)	−1.48	−2.01; −0.94	<.001	−3.64	−4.36; −2.91	<.001	<0.001
Weight loss (%)	−1.2	−1.68; −0.78	<.001	−3.2	−3.90; −2.47	<.001	<0.001
BMI (kg/m^2^)	−0.54	−0.74; −0.34	<.001	−1.32	−1.59; −1.04	<.001	0.036
Waist Cir. (cm)	−2.8	−4.7; −1.0	.005	−7.6	−11.6; −3.5	.001	0.012
FM (%)	−0.5	−1.2; 0.2	.175	−1.0	−2.1; 0.1	.079	<0.001
FM (kg)	−1.0	−2.6; 0.53	.186	−2.5	−4.0; −0.9	.003	0.004
FFM (%)	−0.3	−0.7; 1.3	.533	−0.8	−0.5; 2.0	.213	0.922
FFM (kg)	−0.3	−1.4; 0.9	.649	−1.1	−2.6; 0.4	.150	0.001
Energy intake (kcal/day)	−354	−436; −303	.038	−507	−583; −430	<.001	0.031
Measured REE (kcal/day)	−113	−200; −83	.015	−134	−191; −85	.023	0.016
Predicted REE (kcal/day)	−43	−104; 18	.161	−65	−123; −6.8	.031	0.002
REE corrected for FFM (kcal/day/kg)	−1.46	−2.76; 0.015	.030	−2.34	−3.52; −1.16	<.001	<0.001
RQ	−0.008	−0.058; 0.041	.731	−0.090	−0.162; −0.017	.018	0.744

Abbreviations: BMI, body mass index; FM, fat mass; FFM, fat‐free mass; MD, mean difference; REE, resting energy expenditure; RQ, respiratory quotient; WHR, waist to hip ratio; 95% CI, 95% confidence interval.

### 5.2 Patient acceptability and tolerability using indirect calorimetry

Fifty participants completed the 5‐point Likert scale questionnaire to measure acceptability and tolerability using the ECAL IC with responses ranging from ‘strongly disagree’ (1) to ‘strongly agree’ (5). Participants in both groups reported high levels of acceptability and tolerability for using the ECAL IC after the 24‐week weight loss programme, as evidenced by the overall mean acceptability and tolerability score of 4.5 ± 0.6 and 4.2 ± 0.7, in the INT and SOC groups respectively.

### 5.3 Energy expenditure data analysis

There was an observed decrease in REE from baseline to week 24 (*p* = .004) in both groups after adjusting for baseline body mass index, age, and gender. At baseline, the measured RQ was similar between groups, with INT at 0.97 (±0.03) and SOC at 0.98 (±0.03). After 4 weeks, the RQ value decreased in both the INT (0.84 ± 0.06, *p* <.001) and the SOC (0.91 ± 0.08, *p* <.001) groups compared to baseline. The decrease in RQ was greater in the INT as compared to SOC (*p* <.001) (Supplementary Material). After 8 weeks, the RQ value remained lower in the INT group (0.89 ± 0.06), but the RQ value had increased in the SOC group (0.96 ± 0.05), and the RQ value returned to baseline values. After 12 weeks, RQ was not different between INT versus SOC, with INT at 0.88 (±0.05) and SOC at 0.90 (±0.05) (*p* = .10). After 24 weeks, there was no difference in the RQ between INT and SOC groups (0.91 ± 0.05 vs. 0.92 ± 0.05; *p* = .24).

**FIGURE 3:**
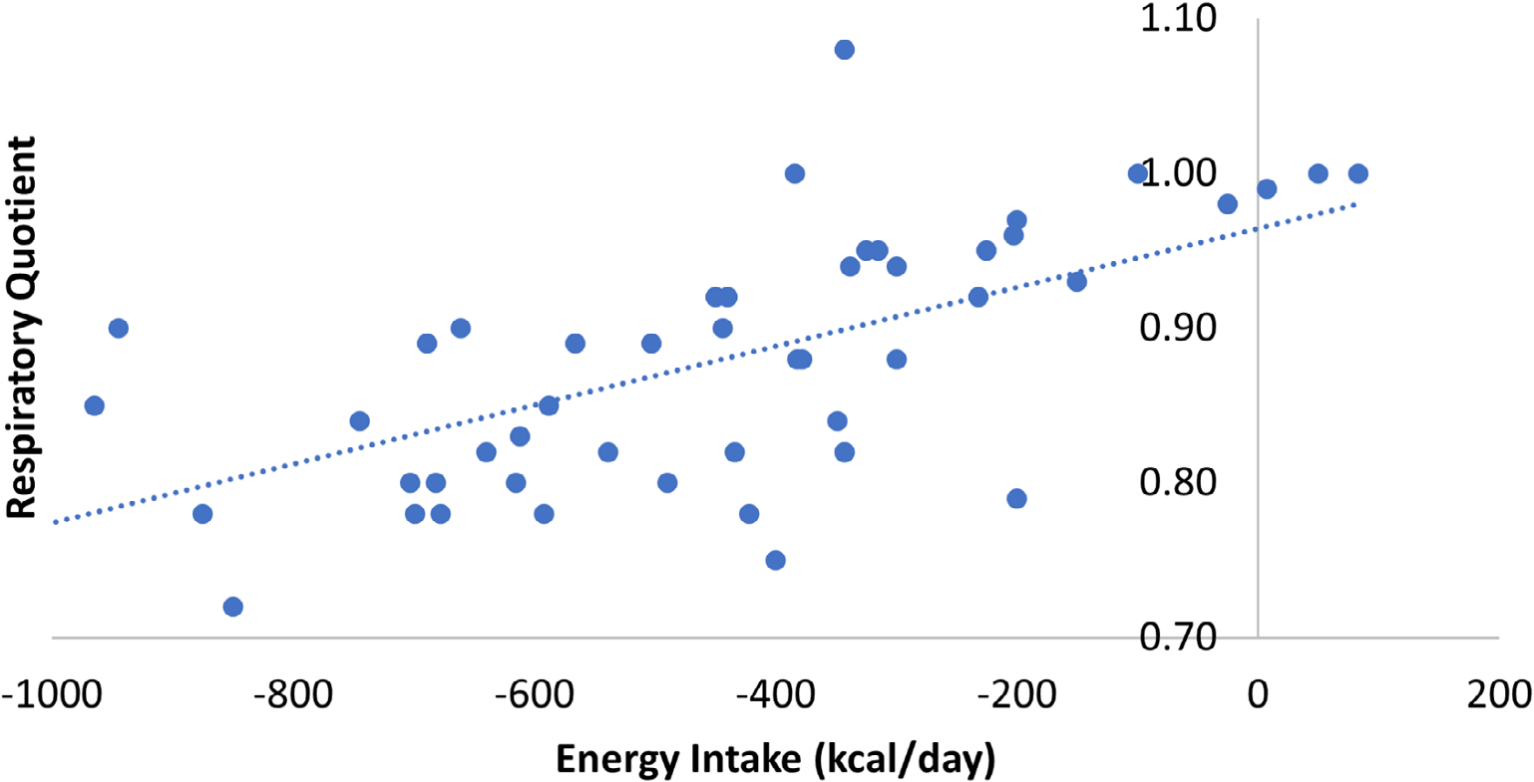
Scatter plot of RQ against the energy intake. Sustained decrease in energy intake positively correlated with reduced RQ value (*r* = 0.42; *p* = .003).

The mean RQ decreased notably with weight loss (Supplementary Material). The RQ decrease was most apparent between baseline and week 12 (MD −0.09; 95% CI −0.11 to −0.06; *p* <.001), during the acute phase of energy restriction. It suggests that prolonged energy restriction may enhance fat oxidation capacity, indicated by a decrease in RQ value. No correlation was observed between measured RQ and the change in body fat (*p* = .74). Sustained decrease in energy intake positively correlated with reduced RQ value (*r* = 0.42; *p* = .003) (Figure 3).

### 5.4 Dietary intake

Based on the returned food diaries, self‐reported energy intake decreased in both the INT (−551 ± 232 kcal/day) and SOC (−357 ± 253 kcal/day) groups between baseline and end of study (Supplementary Material). The decrease in energy intake correlated with weight loss after 24 weeks (*p* <.001).

### 5.5 Activity‐related energy expenditure

The AEE was compared between baseline, week 12, and the end of the 24‐week intervention (Supplementary Material). At baseline, participants in both INT and SOC groups reported similar duration of walking (min/week) (median(IQR)) (INT: 224 (65) vs. SOC: 245 (58); *p* = .072), moderate physical activity (min/week) (INT: 114 (34) vs. SOC: 110 (20), *p* = .676), and vigorous physical activity (min/week) (INT: 10 (19) vs. SOC: 17 (18); *p* = .356). After the 24‐week intervention, participants in the INT group demonstrated a greater increase in moderately intense physical activity (INT 173 (26) vs. SOC 136 (30) min/week; *p* <.001) and vigorous physical activity (INT 165 (215) vs. SOC 79 (111); *p* = .004), but no difference in walking time (INT 465 (125) vs. SOC 484 (105); *p* = .518).

## 6 DISCUSSION

This assessor‐blinded, parallel‐group, randomized controlled trial found that individuals who received personalized biofeedback from real‐time EE and RQ data from portable IC lost more weight compared to those who received standard weight loss advice. The intervention group also demonstrated greater adherence to dietary restrictions and energy goals, along with higher levels of physical activity. The study demonstrated the potential for user biofeedback to offer personalized insights into an individual's energy expenditure, which can be a powerful tool in behaviour‐based weight loss interventions. It also establishes the safety and practical application of tracking real‐time energy expenditure from a portable IC promoting self‐regulation. The provision of biofeedback for weight loss in our study resulted in a greater proportion of the intervention group achieving clinically important weight loss outcomes, specifically achieving a 5% reduction in baseline weight, which is widely recognized as a meaningful threshold for significant health improvements.^12^

### 6.1 Substrate utilization during energy restriction

During energy restriction, substrate utilization shifts towards fat metabolism (either from skeletal muscle stores or release of non‐esterified fatty acids from lipolysis), and the RQ value decreases.^13^, ^14^, ^15^ The knowledge of this may help individuals understand whether energy restriction and higher physical activity levels may influence their body fat oxidation.^16^, ^17^, ^18^ While a lower RQ indicates a higher proportion of fat oxidation, overall changes in body fat mass are determined by the balance of total energy intake and expenditure over time. The variations in REE and non‐exercise activity thermogenesis can obscure the direct relationship between RQ changes and body fat mass reduction.^6^

The absence of a direct correlation between a decrease in RQ and changes in body fat mass highlights the multifaceted nature of metabolic regulation and weight loss. There is considerable inter‐individual variability in the efficiency of fat oxidation.^19^, ^20^, ^21^ Genetic factors, hormonal influences, and differences in muscle fibre composition can all affect how effectively individuals oxidize fat.^6^, ^21^ Indirect calorimetry, can be influenced by factors such as recent physical activity, diet, and stress levels. Exercise programs focusing on lower‐intensity, longer‐duration exercise interventions, which lower RQ, were more effective in reducing body fat compared to high‐intensity, short‐duration activities.^22^

### 6.2 Change in REE during energy restriction

We report a maintenance or rise in REE during the acute phase of energy restriction (weeks 4–12). This may be due to the change in body composition, i.e., an increase in lean muscle mass relative to fat mass, leading to relatively higher REE.^23^, ^24^, ^25^ During energy restriction, metabolic adaptations and hormonal changes (increased levels of thyroid hormones or catecholamines), may result in an unexpected rise or maintenance of REE.^25^, ^26^ Higher protein intake could increase thermogenesis and maintain lean body mass, contributing to a higher REE.^13^, ^27^ This effect can sometimes mask the expected decrease in REE that accompanies weight loss.^28^

Several prospective studies have examined the relationship between future weight change and energy metabolism variability under near‐energy balance conditions.^29^ Evidence from longitudinal studies has suggested a correlation between lower REE and prediction of long‐term weight and fat mass gain.^18^, ^30^, ^31^ Our study, however, with its small sample size, high inter‐individual variability, varying durations of measurement (20–30 min), and differences in physical activity, did not find an association between lower/higher REE and weight loss.

### 6.3 Behaviour change and weight loss

Many individuals find it challenging to consistently follow dietary restrictions and maintain regular exercise routines.^4^ Compensatory metabolic responses, such as reduced REE and increased hunger occur during periods of energy restriction, making it harder to sustain weight loss.^28^ Stress, emotional eating, weight stigma, and lack of motivation can hinder progress.^4^, ^32^ Evidence from systematic reviews have shown that behaviour modification tools can lead to positive, sustained changes in physical activity and dietary adherence, improving weight loss outcomes.^33^, ^34^, ^35^ Considerable research has been conducted on developing and testing specific strategies for maintaining weight loss, but these strategies are not regularly implemented in routine practice. Low intensity interventions have proven to be ineffective,^36^ whereas a systematic review of more intensive interventions providing ongoing behavioural support, in effect extending the behavioural weight loss programmes have found that they enhance weight loss,^37^ improved engagement,^38^ encouraged self‐efficacy, and improved stimulus control.^39^ However, sustained energy restriction and lifestyle interventions often result in only modest long‐term weight loss,^40^ underscoring the need for continuous support and personalized strategies to enhance long‐term weight outcomes.^41^

The success of providing energy expenditure biofeedback for weight loss in people with obesity may be attributed to increased awareness, motivation, and improved self‐regulation.^42^ However, the cost and the logistical challenges of biofeedback interventions may limit their widespread applicability, particularly in resource‐constrained healthcare settings.^43^ We suggest prioritizing this strategy for patients with severe obesity who are preparing for elective surgery to encourage preoperative weight loss and improve postoperative outcomes.^44^, ^45^

Strengths of this trial include a well‐conducted assessor‐blinded randomized controlled design with comprehensive data on anthropometric and metabolic evaluation in all participants recruited from a specialist weight management centre. High adherence and completion rates marked its success. However, challenges like a low recruitment rate due to COVID‐19 impacts, suspended recruitment, and diminished interest hindered reaching recruitment targets. Despite these issues, the trial yielded promising weight loss data from a small but demographically consistent cohort. A notable gap remains in validating the portable ECAL indirect calorimetry, highlighting the need for further studies on its precision and dependability.^29^

## AUTHOR CONTRIBUTIONS

The study investigators JPHW, JZML, and UA were responsible for creating the research question, designing the study, obtaining ethical approval, acquisition of funding, and subsequent data and oversight of the study. JZML, AW, JB, JO, MJ, and AC administered and conducted the investigation. JZML, AW, and JB were responsible for collating the data. JZML and DH performed the statistical analyses with input from JPHW, UA, DJC, and JB; JZML wrote the first draft of the manuscript. All authors helped interpret the data, revised the manuscript, and approved the final version.

## CONFLICT OF INTEREST STATEMENT

We acknowledge Metabolic Health Solutions Pty Ltd, Australia, for sponsoring the ECAL IC devices and providing support for service and calibration of the device. The funding source and funding bodies did not have any input into the design of the study, the collection or analysis of data, the preparation of this manuscript, or the decision to submit this manuscript for publication. JPHW has received research grants from AstraZeneca, Novo Nordisk, Lilly, and Rhythm Pharmaceuticals. JPHW has received lecture fees from AstraZeneca, Boehringer Ingelheim, Napp, Novo Nordisk, and Medscape. UA has received investigator‐led funding from Procter & Gamble. UA has received honoraria from Procter & Gamble, Viatris, Grunenthal, and Sanofi for educational meetings. UA has received funding for attendance at an educational meeting by Daiichi Sankyo and has received an investigator‐led grant from Procter & Gamble.

## Supporting information


**Data S1:** Supporting Information
