# Supplementary material for: Efficacy of providing energy expenditure information to guide weight loss interventions in people with obesity: A randomized controlled trial
Source: Clin Obes. 2024 Sep 17;15(1):e12703. doi: 10.1111/cob.12703 (PMC11706736; doi:10.1111/cob.12703)
Supplement: Supplementary file 1 — Data S1: Supporting Information [file COB-15-e12703-s001.pdf]

# Efficacy of Providing Energy Expenditure Information to Guide Weight Loss Interventions in People with Obesity: A Randomized Controlled Trial.

**Jonathan ZM Lim[1,2,3], Andrew Williams[1], Jamie Burgess[1], James O'Connell[4], Michaela James[4], Andy Cross[1], David Hughes[5], Daniel J Cuthbertson[1], Uazman Alam[1,3,6], John PH Wilding[1,4].**

**1. Department of Cardiovascular & Metabolic Medicine, Institute of Life Course and Medical Sciences, Clinical Sciences Centre, Liverpool Centre for Cardiovascular Science, University of Liverpool, Liverpool, UK**

**2. Diabetes, Endocrinology, and Metabolism Centre, Manchester Royal Infirmary, Manchester University NHS Foundation Trust, Manchester Academic Health Science Centre, UK**

**3. Institute of Cardiovascular Sciences, Cardiac Centre, Faculty of Medical and Human Sciences, University of Manchester and NIHR/Wellcome Trust Clinical Research Facility, Manchester, UK**

**4. Aintree Weight Management Services, Nutrition and Dietetics Therapies, Aintree University Hospital, Liverpool University Hospitals NHS Foundation Trust, Liverpool, UK**

**5. Department of Health Data Science, Institute of Population Health, Faculty of Health & Life Sciences, University of Liverpool, Liverpool, UK**

**6. Centre for Biomechanics and Rehabilitation Technologies, Staffordshire University, UK**

Address for correspondence:

Jonathan ZM Lim
Diabetes, Endocrinology, and Metabolism Centre,
Manchester Royal Infirmary,
Manchester University NHS Foundation Trust,
Manchester Academic Health Science Centre,
Oxford Road
Manchester
M13 9WL
United Kingdom
E-mail: Jonathan.Lim2@mft.nhs.uk

**Figure S1**: Scatter plot of the change in respiratory quotient (RQ) and change in body weight (%) (baseline to week 24). Associations were investigated using Spearman correlation analysis. Decrease in respiratory quotient correlated with the decrease in body weight (r=0.62; P <0.001).

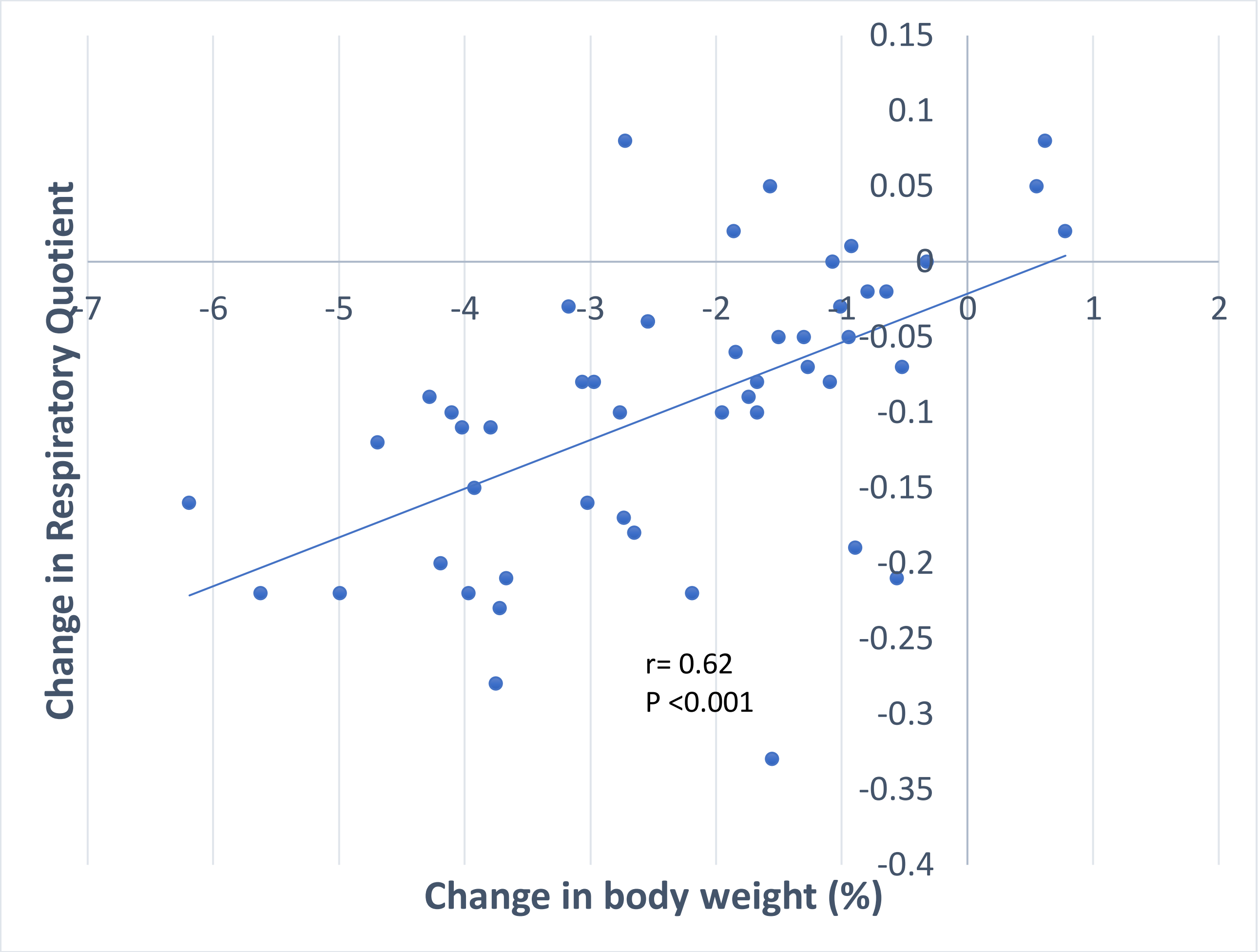

**Figure S2**: Mean RQ values at follow-up visits comparing SOC vs INT. Error bars represents standard error.

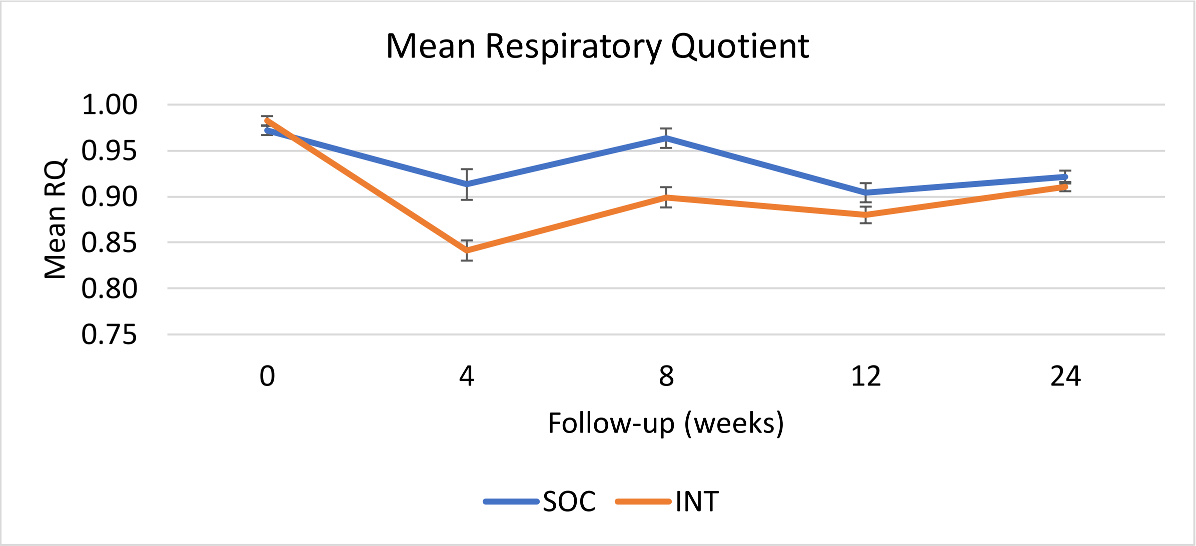

**Figure S3**: Mean REE at follow-up visits between SOC vs INT. Error bars represents standard error.

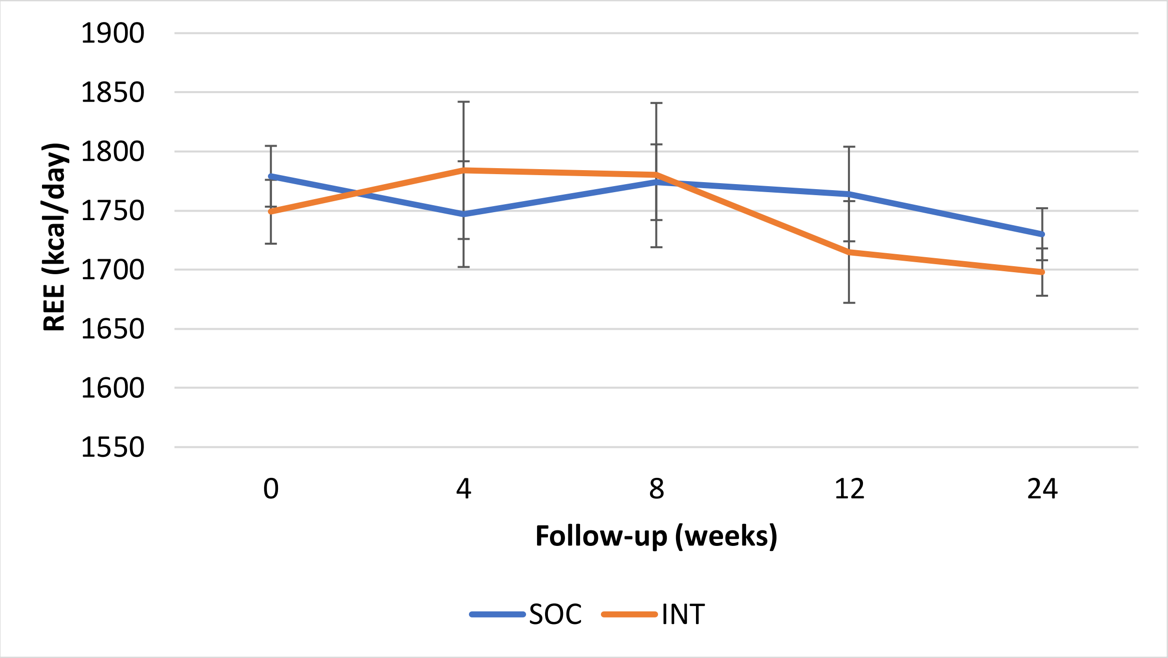

**Figure S4**: Bar chart representing the reported duration of physical activity (walking, moderate or vigorous intensity exercise; minutes/week) at baseline (W0), week 12 (W12) and end of study (week 24) (W24).

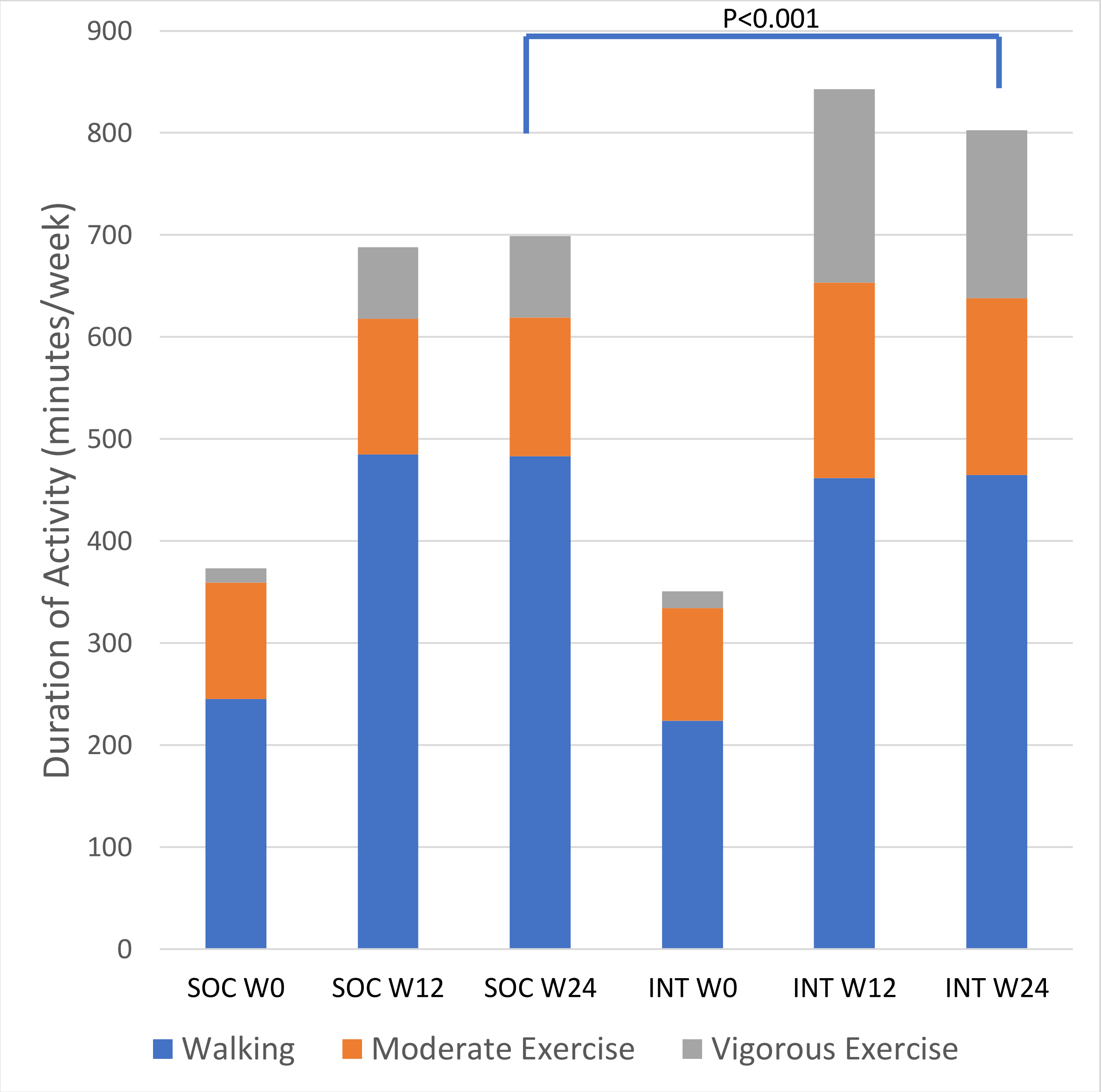

Further Information about the ECAL Indirect Calorimeter can be found on website:
https://www.metabolichealthsolutions.org/ecal-technology/

Introducing the ECAL indirect calorimetry. Video Link on YouTube:
https://www.youtube.com/watch?v=wuAxcBLQ8TU

**Figure S5**: Example of a layperson summary generated from ECAL indirect calorimeter.

# RESTING METABOLIC PROFILE

**Client Name:** [redacted]
**Date of Test:** [redacted]

| | Test Score | Normal Range | Optimal |
|---|---|---|---|
| Resting Energy Production (REP) (kcal) | 1907 | 1697 - 2075 | N/A |
| Resting Energy Production (REP) (kJ) | 7984 | 7105 - 8688 | N/A |
| RQ Score | 1.05 | 0.75 - 0.85 | 0.75 |
| Fat Burning (%) | 0 | 48 - 83 | 80 |
| Glucose Availability (%) | 113.4 | 17 - 52 | 20 |
| Mitochondrial Efficiency (FEO2) (%) | 17.79 | 16 - 17 | < 15 |
| Carbon Dioxide Production (FECO2) (%) | 3.35 | 3.5 - 4.5 | 3.5 |
| Breathing Rate (breaths/min) | 11.27 | 5 - 15 | 5 - 8 |
| Breathing Volume (litres/breath) | 0.90 | 0.4 - 1.0 | 0.50 |

## Anthropometric Data

| | Initial | Previous | Current |
|---|---|---|---|
| Date | [redacted] | [redacted] | [redacted] |
| Time | [redacted] | [redacted] | [redacted] |
| Weight (kg) | 123.6 | 123.7 | 121.6 |
| Height (cm) | 161 | 161 | 161 |
| Waist Circumference | 128 | 128 | 116 |
| Hip Circumference | 135 | 135 | 138 |
| Neck Circumference | - | - | - |
| Waist to Hip Ratio | 0.95 | 0.95 | 0.84 |
| Left mid Bicep | - | - | - |
| Right Mid Bicep | - | - | - |
| Left Thigh | 72 | 72 | 67 |
| Right Thigh | - | - | - |
| Body Fat (%) | 52.9 | 52.9 | 53.4 |
| Body Fat (kg) | 65.4 | 65.4 | 64.9 |
| Active Tissue Mass (%) | 47.1 | 47.1 | 46.6 |
| Active Tissue Mass (kg) | 58.3 | 58.3 | 56.7 |
| Hydration | - | - | - |
| Total Body Number | - | - | - |
| Fasting Period (hours) | 12 | 12 | 12 |

## Practitioner Notes

Fasting, 12 hours. Volume 0.9, RR 11.2
